# Nephroprotective Potential of Human Albumin Infusion: A Narrative Review

**DOI:** 10.1155/2015/912839

**Published:** 2015-06-07

**Authors:** Christian J. Wiedermann, Michael Joannidis

**Affiliations:** ^1^Department of Internal Medicine, Central Hospital of Bolzano/Bozen, Lorenz-Böhler Street 5, 39100 Bolzano, Italy; ^2^Interdisciplinary Medical Research Institute South Tyrol (IMREST), Lorenz-Böhler Street 5, 39100 Bolzano, Italy; ^3^Division of Intensive Care and Emergency Medicine, Department of Internal Medicine I, Medical University of Innsbruck, Anich Street 35, 6020 Innsbruck, Austria

## Abstract

Albumin infusion improves renal function in cirrhosis; however, mechanisms are incompletely understood. In clinical practice, human albumin is used in various intensive care unit indications to deal with a wide range of problems, from volume replacement in hypovolemic shock, or sepsis, to treatment of ascites in patients with liver cirrhosis. Against the background of the results of recent studies on the use of human albumin in septic patients, the importance of the natural colloid in these critically ill patients is being redefined. In addition to the hemodynamic effects of administration of human albumin impacting on sympathetic tone, attention is being paid to other effects in which its pharmacodynamics is associated with the physiological importance of endogenous albumin. The morbidity and mortality data discussed in this paper support the importance of both the hemodynamic and the pharmacological effects of the administration of human albumin in various indications. The contribution that human albumin could make towards the maintenance of renal function in the course and treatment of severe sepsis and cirrhosis of the liver is the subject of this narrative review.

## 1. Introduction

Criteria for ideal volume substitute, particularly its safety aspect, are increasingly becoming the subject of discussion. High-quality, large-scale studies have shown the risks associated with preparations of hydroxyethyl starch (HES). This has led to moving away from artificial colloids and paved the way for the development of lower-risk crystalloids. The update on guidelines reflects this development: The International Surviving Sepsis Campaign [1] recommends that, for initial volume substitution in severe sepsis and septic shock, crystalloid preparations should be used first and not HES. Evidence bases for this recommendation are the studies VISEP, CRYSTMAS, 6S, and CHEST [2–5], which showed a trend in the HES groups [2, 3, 5] or significance [4] for higher mortality and the need to employ renal replacement therapy significantly more frequently. These are hard clinical endpoints in stark contrast to small trial results available for efficacy and safety of artificial colloid infusions using nonvalidated surrogate markers. If, in critically ill ICU patients, crystalloids alone are not sufficient and a need for colloid is assumed, the administration of natural albumin may be considered according to current recommendation [1].

In patients with cirrhosis of the liver, effective arterial hypovolaemia is the pathophysiological characteristic especially when the stage of disease is advanced or decompensated. Since the main physiologic function of albumin is related to its oncotic activity maintaining colloid osmotic pressure, infusion of albumin can achieve effective plasma volume expansion. This is supported by its prolonged half-life in the intravascular compartment. Pharmacodynamic effects may also include nononcotic properties such as ligand-binding and transport of various molecules in addition to antioxidant and anti-inflammatory actions. These functions may play a role in various clinical scenarios including renal function and the development of sepsis in patients with cirrhosis [6]. This review discusses the potential benefits of albumin infusion for renal function in patients with cirrhosis of the liver.

## 2. Human Albumin Infusions in Sepsis

Albumin infusion in volume therapy has a place if persistent volume effect is to be achieved with lower infusion volumes and artificial colloids, which according to approved indications may not be used in intensive care unit patients for safety reasons. Since crystalloids in fluid resuscitation of sepsis patients do not lead to stable hemodynamics, the natural colloid albumin should be additionally used according to the guidelines [1]. In the randomized, double-blind SAFE study, it was observed that, in the ICU, general fluid replacement with 4% albumin solution compared with 0.9% NaCl solution was safer [7–9]; in the predefined subgroup of patients with severe sepsis, the mortality rate in the albumin group was lower than in the group receiving saline solution (risk of mortality reduced by 29%, *p* < 0.05), and it was found that particularly those derived greater benefit whose serum albumin levels had been increased to above 25 mg/dL.

Since albumin in plasma is mainly responsible for the colloid osmotic pressure and, as the main carrier protein, has antioxidant and anti-inflammatory properties, the therapeutic efficacy of albumin can be enhanced by correcting low levels of serum albumin during volume therapy; that is, hypoalbuminemia is corrected by raising serum albumin levels to ≥30 g/L [10]. The ALBIOS study, with the goal of maintaining the level of albumin at 30 g/L during patient stay in the ICU or up to 28 days after randomization, is particularly relevant in this context [11]. ALBIOS was an open-label, randomized, and controlled study in which a comparison was made of the efficacy of crystalloid saline without and with administration of 20% albumin solution in 100 intensive care units in Italy with a total of 1818 patients with severe sepsis and septic shock. The primary endpoint of mortality was defined at 28 days and the secondary endpoints included mortality at 90 days, the incidence of organ dysfunction, and length of stay in the ICU and in the hospital. Neither after 28 nor after 90 days was there a significant difference in survival in the overall population; in the subgroup of patients with septic shock which included more than 1,300 people, in the albumin-treated group there was a significant survival advantage after 90 days (42.6% in the human albumin group and 48.4% in the control group, *p* = 0.03); however, this post hoc analysis was not predefined and needs confirmation in further studies.

The pooled analysis of mortality data from the three largest studies of volume therapy with human albumin in sepsis, namely, SAFE, ALBIOS, and the EARSS (not yet published study) confirmed that administration of the natural colloid can significantly reduce mortality in patients with severe sepsis or septic shock (*p* = 0.046) [12]. The meta-analysis by Patel et al. [13] failed to confirm the efficacy of human albumin solutions in sepsis patients, but this might be due to the fact that its mortality data included a number of “high-risk-of-bias” studies such as attrition bias in EARSS, one of the three big studies [13], inclusion of studies at high risk of falsification [14], and double counted mortality data [15]. It is perilous to conclude from a hypothesis test that albumin is not effective, especially when the test rests on a bias-prone result. In contrast, the meta-analysis of Rochwerg et al. [16] came to the conclusion that when volume therapy with human albumin solutions is performed, lower sepsis mortality is achieved. Problems about albumin that remain unclear include which concentration (4% to 5% versus 20% to 25%) and what dose of albumin should be used and if use of balanced versus unbalanced solutions matters.

## 3. Nephroprotection by Albumin

The ALBIOS data showed that, in the subgroup of patients with septic shock, treatment with albumin resulted in not only reduction in mortality but also favorable negative fluid balance at an earlier stage and hemodynamic stabilization was achieved more frequently in the first 24 hours; also the need for a vasopressor therapy was shortened [11]; however, the need to employ renal replacement therapy was not significantly affected.

### 3.1. Improving Microcirculation by Albumin

Recently it was discovered that the surface layer of endothelial cells, which is composed of plasma proteins and glycocalyx, plays an important role in the vascular barrier function, in edema development and the leukocyte-endothelium interaction. In studies using different models it was shown that human albumin improved the endothelial integrity by substantially protecting the glycocalyx of endothelial cells [17].

According to other data from basic research, extracellular heme leads to concentration-dependent organ failure, because, in combination with TNF and/or reactive oxygen species, it is highly cytotoxic and reduces microcirculation [18]. Albumin prevents these two detrimental effects of heme, while, for instance, administration of HES has no effect on the disorder of the microcirculation [19]. In addition to maintaining the colloid osmotic pressure, the physiological functions of albumin such as the transport of water-insoluble substances in the blood, its buffering properties in acid-base balance, and its anti-inflammatory and antioxidative effects [6, 20–22] probably support its favorable effects on hemodynamics and might help cell and organ function in severely ill patients (Table 1).

### 3.2. Hypoalbuminemia as a Risk Factor for Acute Kidney Injury

For septic patients, renal function is prognostically important. The relationship between hypoalbuminemia and the risk of developing septic renal failure has been investigated in a meta-analysis of clinical observational studies describing the relationship between serum albumin and the incidence of acute kidney injury (AKI) using multivariate analysis. In addition, the impact of lower serum albumin levels on mortality in patients with AKI was analyzed [23]. Eligible studies were identified by various search methods and meta-analytic procedures were used to estimate adjusted odds ratios (ORs) using a random effects model. Seventeen trials involving 3917 patients were included: 11 studies (six with surgical or intensive care unit patients and 5 patients from other hospital departments) enabled assessment of the influence of serum albumin on the AKI incidence and 6 studies enabled the relationship between lowering of the serum albumin levels and mortality in patients who had developed AKI. Hypoalbuminemia was confirmed as an independent predictor for both developing AKI and death after development of AKI. With every decrease of serum albumin by 10 g/L, the risk of AKI increased by 134%; the pooled OR for AKI was 2.34 with a 95% confidence interval (CI) of 1.74 to 3.14. In patients who developed AKI, with each decrease of serum albumin by 10 g/L, mortality (95% CI 1.51 to 4.05, pooled OR 2.47) increased by 147%. This meta-analysis showed that hypoalbuminemia is a significant independent predictor of both AKI and death after AKI. Serum albumin determination could be useful in identifying patients at increased risk for developing AKI or death after AKI [23]. It is therefore reasonable to investigate whether the correction of hypoalbuminemia by infusion of human albumin could not lead to a reduction of AKI in such patients.

### 3.3. Mechanisms of Albumin-Mediated Renal Protection

Administration of the natural human albumin as a colloid is not nephrotoxic [3], as opposed to artificial colloids such as HES. Through various signal transduction pathways, endogenous albumin is involved in maintaining the integrity and function of the proximal tubule cells [24]. Against this background, the implementation of prospective randomized controlled trials on renal protection with exogenous human albumin in patients with hypoalbuminemia seems more than justified. In addition to the proliferation-regulating effect on tubular cells, a number of other pleotropic properties of albumin might also be effective.

#### 3.3.1. Mitigation of the Nephrotoxicity of Medications

In a cohort study investigating the incidence and risk factors of amikacin-induced nephrotoxicity, 104 patients were treated for at least 36 hours with intravenous amikacin and prospectively followed up. Serum creatinine was determined every second day and plasma concentrations of amikacin were determined 48 and 96 hours after treatment. Patients with other risk factors for AKI were excluded [25]. Ten of 104 patients developed nephrotoxicity (9.6%). In a logistic regression model, the serum albumin concentration was the most powerful predictor of nephrotoxicity of aminoglycoside therapy. The lower the serum albumin concentration, the higher the risk of toxicity. The mean serum albumin concentration in patients with renal toxicity was 26 g/L, while it was 35 g/L in the group of patients without toxicity. There was no difference between the two groups in age, sex, diagnosis, renal function, and amikacin doses. In addition, low serum albumin concentrations were also associated with increased amikacin trough levels in plasma, which also was independent of age, sex, initial renal function, and dose of amikacin administered [25]. It can be deduced that the concentration of serum albumin is the most powerful predictor of amikacin-induced nephrotoxicity. This further suggests that the potential nephrotoxicity of other drugs might also be associated with plasma serum albumin concentrations.

#### 3.3.2. Restoration of Balanced Net Fluid Balance

Fluid retention is a common complication in patients with diseases requiring treatment in intensive care unit. It arises from infusion of large volumes of fluid carried out in the context of volume replacement therapy and is usually worsened by hypoalbuminemia. Edema resulting from fluid retention is responsible for delayed weaning from ventilation and associated with various organ complications and increased mortality. Administration of diuretics for the restoration of a balanced net fluid balance is considered to be the standard treatment.

In patients with nephrotic syndrome who develop macroalbuminuria and also edema, there is often a reduction of the natriuretic effect of the loop diuretic furosemide. In a double-blind, placebo-controlled study of nine nephrotic patients administered standardized sodium chloride, the following intravenous administrations were performed over 60 minutes in random order on three separate days [26]: (1) 60 mg furosemide in 200 mL placebo solution, (2) 60 mg furosemide in 200 mL of a 20% solution of human albumin, or (3) placebo in 200 mL of a 20% solution of human albumin. As endpoints of the study, the urine volume and urinary excretion of sodium, albumin, and furosemide, renal hemodynamics, and the plasma concentration of the atrial natriuretic factor were measured. The sole administration of furosemide caused a significantly greater urinary volume and urine sodium excretion than when only human albumin was administered. The simultaneous administration of furosemide and human albumin caused a significant increase in furosemide effect, and plasma levels of atrial natriuretic factor, serum albumin concentrations, and the values of urinary albumin excretion increased when the infusion contained human albumin. The administration of human albumin is therefore able to potentiate the effect of furosemide in patients with nephrotic syndrome due to changes in renal hemodynamics [26].

Hypoproteinemic patients with acute lung injury tend to increase fluid accumulation in the lungs and may respond clinically better to a combination of albumin with furosemide than to the diuretic alone. In another randomized, double-blind, placebo-controlled multicenter study of 11 medical, surgical, or trauma ICUs, 40 ventilated patients with acute respiratory distress syndrome (ARDS) were investigated, whose serum protein concentrations were less than 60 g/L; hemodynamically instable patients or those with significant renal or hepatic failure were excluded [27]. The participants were randomized to a 72-hour treatment in two groups: furosemide with albumin or furosemide with placebo. Fluid balance and normalization of serum protein concentration and the oxygenation situation were compared. There were no differences in the baseline characteristics of the two groups. Patients receiving albumin showed better oxygenation and better serum protein concentrations than the control group and significantly lower fluid retention (more net negative fluid balance, minus 5480 versus minus 1490 mL at day 3; *p* < 0.05); control patients developed more frequently hypotension and had less vasopressor-free ICU days. This was reflected in the corresponding differences in the extent of organ failure at end of study [27].

In a further study investigating whether administration of human albumin to correct hypoalbuminemia in intensive care units can also have positive effects on various organ functions, the consequences of human albumin administration for the net fluid balance were, among others, tested in a surgical-internist mixed population of critically ill patients [28]. One hundred adult patients with a serum albumin concentration of 30 g/L or less were randomly assigned to two groups; one group was treated with 300 mL of 20% albumin solution on day 1 followed by 200 mL/day as long as the serum albumin concentration remained below 31 g/L, and the other was the control group not receiving albumin treatment. The primary endpoint was the effect of albumin on organ function, assessed by the SOFA score from day 1 to day 7. At baseline, the two groups of 50 patients each were comparable with respect to age, sex, serum albumin concentration, and APACHE II scores. Organ function improved in the albumin group better than in the control group (*p* = 0.026). Although the use of diuretics was identical in both groups, fluid retention in the control group was almost three times as high as in the albumin group (1679 versus 658 mL, *p* = 0.04) [28]. Although comparable diuretics were used, correction of hypoalbuminemia by administration of human albumin solutions resulted in less pronounced fluid retention.

The addition of albumin to furosemide therapy of ARDS therefore improved hypoproteinemic patients because of a negative net fluid balance, oxygenation parameter, and hemodynamic stability. Also in other clinical situations in patients with hazardous fluid retention, protective negative balance could be more easily achieved with human albumin infusions than without [27–29].

## 4. Fluid Overload as Renal Risk Factor

Positive fluid balance increases the risk for the development of intra-abdominal compartment syndrome and increased renal venous pressure. Renal interstitial edema, which develops because of increased venous pressure within the encapsulated organ, results in the impairment of the filtration performance that contributes to changes in the tissue architecture and thus the blood flow, metabolism, and diffusion changes. Normal intercellular communication mechanisms are lost resulting in significant deterioration of organ function. Even a fluid overload of 5–10% body weight in critically ill patients is associated with prolonged mechanical ventilation, impaired gas exchange, and poorer postoperative results in wound healing and blood clotting [30].

The association between systemic haemodynamics and new or persistent AKI in severe sepsis was studied retrospectively in 137 septic surgical ICU patients; of these, 69 had new or persistent AKI [31]. Patients with AKI had higher central venous pressure values that were associated with the risk of developing new or persistent AKI even after adjustment for fluid balance and positive end-expiratory pressure level. A linear relationship between the two was observed suggesting a role of venous congestion in the development of AKI.

That increase in renal venous pressure may directly cause sodium retention in the kidney which was described already more than 25 years ago [32]. Thus, a vicious cycle could be triggered because sodium retention causes expansion of the plasma volume and thus a further increase in venous pressure. The sequence of events described above is likely to be crucial for the pathophysiology of chronic right heart failure and may also be relevant for the course of other edematous states, such as cumulative net positive fluid balance after initial generous fluid resuscitation in septic shock. Volume therapy is often performed in patients who not only have a hemodynamic risk factor for AKI but also have other risk factors. The potentially negative impact of excessive fluid therapy is now being increasingly recognized [33]. Particularly in patients with AKI, excessive administration of salt and water predisposes to organ dysfunction, impaired wound healing, and nosocomial infections [34]. In these patients, the excretory function* a priori* is already impaired. As a consequence, additional interstitial edema delays the functional recovery of the kidney. Therefore, conservative strategies in volume therapy are being increasingly advocated [33].

### 4.1. Conservative Strategies in Volume Therapy

The challenge in volume therapy is that while volume resuscitation is needed to restore hemodynamics, this can easily lead to tissue edema, which causes further deterioration in organ dysfunction already present. Conservative strategies aim to achieve a net negative fluid balance once hemodynamic stabilization is achieved. Particularly in patients with AKI, if too much fluid was removed with diuretics or extracorporeal therapy, care must be taken to prevent redevelopment of hypovolemia and renal hypoperfusion. Accurate assessment of fluid status and the careful definition of the targets of volume therapy are thus of particular clinical importance [35]. The success of conservative strategies in fluid management was first seen in the treatment of ARDS patients [36]. Similar randomized, controlled trials in patients with AKI are currently being carried out.

#### 4.1.1. Maintenance of Glomerular Filtration by Human Albumin

It has been shown that the nephroprotective potential of human albumin solutions has hemodynamic and pharmacological effects that act against loss of glomerular function that might occur in various clinical treatment situations due to very different pathophysiological mechanisms.

The influence of intraoperative management on the function of transplanted cadaver kidneys was studied in 438 transplant recipients [37]. The most important factors influencing the frequency of delayed graft function, 1-year graft survival, and overall mortality were the cold ischemia time, duration of surgery, age of the recipient, and the intraoperative administration of human albumin. This makes the intraoperative administration of human albumin one of the most important prognostic factors. A higher dose of albumin induced more and earlier urine production, and after one week, the serum creatinine was 3.4 and 5.8 mg/dL, respectively, in the two groups; the median glomerular filtration rates on days 1 and 7 were significantly different (47 and 28 mL/min, respectively, at higher albumin dose and 33 and 21 mL/min, respectively, at a low albumin dose). Similar differences were noted for the frequency of delayed graft function, primary graft failure, and graft survival at 1 year [37]. It is likely that the intravascular volume expansion with higher doses of albumin was responsible for a faster recovery of circulation and reducing the hypoxia-induced damage of the graft.

Interleukin-2 (IL-2), which is occasionally used in oncological therapy, induces a pathological increase in capillary permeability, manifested by hypotension, tachycardia, and oliguria often seen in septic shock. A prospective randomized study made a comparison of crystalloid and colloid fluid resuscitation in such patients [38]. All patients received maintenance crystalloid fluid and were then randomized to receive crystalloid (0.9% NaCl) or colloidal (5% human albumin) fluid as bolus to maintain vital signs and urine production. Patients who did not adequately respond to administration of fluid bolus were additionally treated with dopamine for oliguria and/or phenylephrine for hypotension. Among a total of 107 patients, as expected, hypoalbuminemia developed more often in the group of patients receiving crystalloid fluid compared to those under therapy with colloids, and despite successful correction of various hemodynamic parameters, oliguria developed in these patients significantly more frequently [38].

The dependence of the function of the glycocalyx on albumin described in chapter 3.1 is likely to play an important role in the development of pathological increase in interleukin-2-induced capillary permeability [39]. The decrease in plasma concentration of albumin is accompanied by a loss of glycocalyx and increases fluid extravasation in animal models [40]. Albumin experimentally protects against the loss of glycocalyx of the capillary surface in ischemia-reperfusion models [17], reduces the damage to the glycocalyx, and facilitates its restoration in hemorrhagic shock [41].

## 5. Clinically Significant Renal Protection by Human Albumin

In the ALBIOS study for severe sepsis or septic shock, no significant difference in renal morbidity was observed between patients treated exclusively with crystalloids and those who additionally received human albumin. Patients in the human albumin group not only achieved more frequently hemodynamic stabilization within the first 24 hours but also had prognostically favorable negative fluid balance [11].

### 5.1. Use of Albumin in Patients with Cirrhosis

Acute kidney injury (AKI) as a complication of cirrhosis is seen in up to 20 percent of patients hospitalized with portal hypertension and portosystemic collaterals both contributing to systemic and splanchnic vasodilatation as the most characteristic consequence. Despite compensatory stimulation of the renin-angiotensin-aldosterone system, the sympathetic nervous system, and the nonosmotic release of antidiuretic hormone, reduction in “effective” arterial blood volume results in sodium retention and a hyperdynamic circulatory state with increased intravascular volume. As cirrhosis progresses, these compensatory adaptions, however, become inadequate. Effective blood volume further decreases with additional activation of vasoconstriction preferentially in renal and central nervous system blood vessels. Normally, decreased renal perfusion activates reflex autoregulation to keep blood flow constant irrespective of fluctuations in systemic blood pressure; however, below a mean arterial pressure of 65 mmHg, renal blood flow starts to decline in proportion to perfusion pressure. Renal hypoperfusion, therefore, is seen as a typical occurrence of advanced cirrhosis, which is responsible for a fall in renal filtration and may also contribute to the development of ischaemic injury if chronically present. On the basis of this low-grade renal hypoperfusion, structural kidney damage with filtration failure and AKI develops more easily when exposed to additional shifts of intravascular volume or nephrotoxic medications. Acute intravascular volume shifts are characteristic of gastrointestinal haemorrhage and large-volume paracentesis (defined as removal of more than 4-5 L of fluid). Numerically, the outpatient use of diuretics and the occurrence of lactulose-associated diarrhoea are responsible for the majority of AKI development in cirrhotics. Vasodilatation may also be caused by inflammatory mediators of spontaneous bacterial peritonitis (SBP) and by its potentially nephrotoxic antibiotic treatment. Although inflammatory kidney conditions may be present in cirrhotic patients, intrinsic renal diseases contribute to AKI of cirrhosis in a minority of cases only. In the majority it is renal hypoperfusion that accounts for more than two-thirds of AKI and will show improvement with volume expansion.

In view of the hemodynamic and renal characteristics achieved by human albumin administration, it can be assumed that the incidence of cardiovascular disorders after large-volume paracentesis in patients with liver cirrhosis can be better prevented by human albumin infusions than by infusions of other solutions. Treatment alternatives to albumin, such as artificial colloids and vasoconstrictors, have been extensively analyzed in this situation. A recent meta-analysis therefore investigated whether morbidity and mortality differ when after paracentesis, patients received human albumin rather than other alternative infusions [42]. The meta-analysis comprised 1,225 patients in 17 randomized trials in which patients with significant ascites were treated with albumin infusions. Primary endpoints were the development of circulatory dysfunction and/or hyponatremia after large-volume paracentesis and patient mortality. Compared to alternative treatments, the infusion of human albumin reduced the incidence of hyponatremia and circulatory disorders as well as the mortality rates. This meta-analysis demonstrates that human albumin administration reduces morbidity and mortality after aspiration of ascites better than alternative treatments for restoring fluid and electrolyte balance [42].

**Figure 1:**
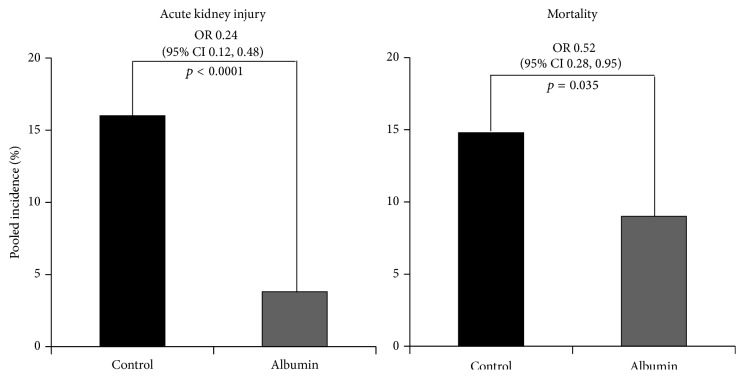
Reduction in the frequency of occurrence of acute kidney injury and death in volume therapy with hyperoncotic human albumin (20% or 25%) compared to control treatment with hypooncotic solution (crystalloids, 4% or 5% human albumin solution) or no infusion. OR, odds ratio; CI, confidence interval. Data are from Wiedermann et al. [43].

The clinical superiority of human albumin after large-volume paracentesis compared to alternative volume replacement could also be due to the renal side effects of volume therapy with artificial colloids. So it was thought that the hyperoncotic properties of infusion solutions and not their pharmacology were responsible for the development of AKI in the critically ill including patients with cirrhosis. Therefore, a meta-analysis of randomized controlled trials compared infusion of hyperoncotic human albumin or HES solutions with hypooncotic solutions [43]. The incidence of AKI was the primary endpoint. Eleven randomized trials involving a total of 1,220 patients were included in the meta-analysis, of which 7 studies were on hyperoncotic human albumin and 4 on hyperoncotic HES. The clinical indications for infusions were ascites, surgery, sepsis, and spontaneous bacterial peritonitis. Hyperoncotic human albumin reduced the incidence of AKI by 76% (OR 0.24; CI 0.12 to 0.48, *p* < 0.0001), while the onset of AKI after hyperoncotic HES significantly increased by 92% (OR 1.92, CI 1.31 to 2.81, *p* = 0.0008). Analogous results were observed also for mortality (Figure 1). This meta-analysis supports the hypothesis that hyperoncotic colloid solutions* per se* do not cause AKI but that the kidney effects are colloid-specific, human albumin is nephroprotective, and HES is nephrotoxic.

In patients with cirrhosis and spontaneous bacterial peritonitis (SBP), the decline of renal function increases mortality in spite of correct administration of nonnephrotoxic antibiotics. Since it was observed that human albumin infusions in patients with SBP improve kidney function and reduce mortality, this hypothesis was also tested in a meta-analysis of randomized controlled trials [44]. Four studies with a total of 288 patients were included in the analysis. In one study, the administration of human albumin was compared with artificial colloid, and in three studies treatment was compared with human albumin-free infusion. All patients received antibiotics. The incidence of renal dysfunction was 31% in the control groups compared with 8% in groups treated with human albumin. This nephroprotective effect of human albumin in SBP was also associated with a significant reduction in mortality [44].

## 6. Conclusions and Future Directions

Hypoalbuminemia is associated with increased risk for the development of AKI and a fatal outcome of AKI. The reduction of circulating albumin levels could have a negative effect on the successful maintenance of adequate renal function in seriously ill patients for the following reasons:

- Albumin not only is primarily responsible for the colloid osmotic pressure in plasma and thus indirectly important for maintenance of kidney function but also has pleiotropic physiological effects including positive effects on vessel wall integrity.
- Administration of human albumin facilitates the achievement of negative fluid balance in hypoproteinemia and in diseases or conditions promoted by edema.
- Infusion of human albumin helps maintaining glomerular filtration via hemodynamic and oncotic mechanisms.
- Fluid resuscitation with human albumin is not nephrotoxic in contrast to artificial colloids.
- Biological plausibility, freedom from nephrotoxicity (safety), and reduction of renal morbidity in liver cirrhosis (effectiveness) speak for the nephroprotective efficacy of human albumin.

The nephroprotective potential of human albumin is supported by its unique pharmacodynamic properties (Table 2). Further clinical studies must show in which clinical indications beyond hepatology this property can be particularly useful as also in what populations albumin could be harmful such as after traumatic brain injury [45]. Better understanding from ongoing randomised controlled trials of the clinical role of iatrogenic hyperchloraemic acidosis induced by 0.9% sodium chloride infusion will also contribute to the clarification of whether or not colloids dissolved in balanced versus unbalanced solution makes a difference.

## Figures and Tables

**Table 1:** Effects of human albumin infusion unrelated to oncotic pressure [6, 17, 19–22].

Effect	Mechanisms
Antioxidation	Increase of plasma thiols

Anti-inflammation	(i) Modulation of cytokine actions (ii) Protection of glycocalyx

Modulation of nitric oxide	—

Ligation of endotoxin	—

Pharmacokinetic and pharmacodynamic effects	(i) Transport of drugs (ii) Modulation of drug effects

**Table 2:** Pharmacodynamic properties of human albumin with nephroprotective potential.

Mechanism	Reference
Mitigation of the nephrotoxicity of medications	[25]
Restoration of balanced net fluid balance	[26–29]
Protection against loss of glycocalyx	[17, 41]
Maintenance of glomerular filtration	[37–39]
